# Predicting Falls and When to Intervene in Older People: A Multilevel Logistical Regression Model and Cost Analysis

**DOI:** 10.1371/journal.pone.0159365

**Published:** 2016-07-22

**Authors:** Matthew I. Smith, Simon de Lusignan, David Mullett, Ana Correa, Jermaine Tickner, Simon Jones

**Affiliations:** 1 Department of Clinical and Experimental Medicine, University of Surrey, Guildford, United Kingdom; 2 Inner North West London Integrated Care Programme, London, United Kingdom; Wayne State University, UNITED STATES

## Abstract

**Introduction:**

Falls are the leading cause of injury in older people. Reducing falls could reduce financial pressures on health services. We carried out this research to develop a falls risk model, using routine primary care and hospital data to identify those at risk of falls, and apply a cost analysis to enable commissioners of health services to identify those in whom savings can be made through referral to a falls prevention service.

**Methods:**

Multilevel logistical regression was performed on routinely collected general practice and hospital data from 74751 over 65’s, to produce a risk model for falls. Validation measures were carried out. A cost-analysis was performed to identify at which level of risk it would be cost-effective to refer patients to a falls prevention service. 95% confidence intervals were calculated using a Monte Carlo Model (MCM), allowing us to adjust for uncertainty in the estimates of these variables.

**Results:**

A risk model for falls was produced with an area under the curve of the receiver operating characteristics curve of 0.87. The risk cut-off with the highest combination of sensitivity and specificity was at p = 0.07 (sensitivity of 81% and specificity of 78%). The risk cut-off at which savings outweigh costs was p = 0.27 and the risk cut-off with the maximum savings was p = 0.53, which would result in referral of 1.8% and 0.45% of the over 65’s population respectively. Above a risk cut-off of p = 0.27, costs do not exceed savings.

**Conclusions:**

This model is the best performing falls predictive tool developed to date; it has been developed on a large UK city population; can be readily run from routine data; and can be implemented in a way that optimises the use of health service resources. Commissioners of health services should use this model to flag and refer patients at risk to their falls service and save resources.

## Introduction

Falls are the leading cause of injury in older people and are expensive for healthcare systems. A third of people over the age of 65 have a fall each year, increasing to 50% in the over 80’s [1,2]. In addition to the human cost of falling (distress, pain, injury, loss of confidence, loss of independence, and mortality), falls are estimated to cost the National Health Service (NHS) upwards of £2 billion a year [3]. Reducing the number of falls could reduce financial pressures on stretched health service budgets. Risk factors for falling in the elderly are extensive [4]. Falls services use risk tools to identify high risk individuals who require an intervention. Many tools exist; however, most use a combination of functional assessment and risk scoring based on known risk factors, such as The Falls Risk Assessment Tool (FRAT) [5], which is the most commonly used assessment tool in UK falls services [6]. These case-finding tools vary widely in performance and little is known about their cost-effectiveness [7].We have previously published a falls risk model produced from general practice data [8]. However, 60% of those that fall present to hospital [9], meaning that all falls may not be accurately represented by general practice data alone. We carried out this research to develop a falls risk model, using routine primary care and hospital data to effectively identify those at risk of a fall, and applied a cost analysis that would enable commissioners of health services to identify those in whom savings can be made through referral to a falls prevention service.

## Methods

### Literature review

We carried out a literature review, searching Medline, PubMed and Cochrane libraries for papers concerning falls interventions, cost of falls interventions, and falls risk modelling. For falls interventions, the terms accidental falls/fractures/hip fractures/hip injuries AND accident prevention were searched. For the cost of falls and interventions, the terms accidental falls/ fractures/hip fractures/hip injuries AND accident prevention AND cost and costs analysis were used. For risk modelling, the terms accidental falls/fractures/hip fractures/hip injuries AND risk modelling/risk assessment/risk were searched. All searches were limited to English language full text articles and ‘aged 65 and over’.

### Study Population

Data for this study were collected as part of an integrated care pilot (ICP) study [10]. These data were routinely collected information from 130 general practice databases across North West London. We set the index date to January 31^st^ 2013, so that we had a year of data before, to build the predictive model, and an after period during which we could predict falls. The total registered population was 656902 patients, of whom 74751 met the eligibility criteria of age 65 years or over, and being registered with their general practice for a year prior to and following the index date. As data were extracted for the whole practice population in each of the 130 practices, there were no exclusions that might have resulted in selection bias being applied. These general practice data were collated with hospital data from three acute providers delivering secondary care to this population. Data collection was performed over a 2.5 year period between September 2011 and February 2014.

### Risk Factors

Many potential risk factors and predictors for falls were identified for inclusion in this study. These were selected from variables extracted as part of the Integrated Care Pilot. Variables were selected if there was evidence suggesting that there was an association with or had the potential to predict falls. Patient factors associated with an increased risk of falling and therefore considered for the model included increasing age [1,2,11,12], female sex [11,13], living alone and disability [14], poor mobility and balance [2,12,15], weight loss/low body weight [12,14], and poor vision [12,16]. Smoking status, vulnerability, and deprivation were also considered as potential risk factors.

Recent healthcare utilisation is a recognised risk factor for falling. Therefore, recent inpatient episode, recent non-elective admission (and the number of non-elective admissions), recent outpatient visit, and a recent accident and emergency attendance (along with the number of attendances) were considered as potential predictors of falls. Previous fracture [12] and previous fall [1,13,16–18] also have a strong evidence base for predicting further falls.

Chronic disease in itself is a well-recognised risk factor for falling, and the risk increases with increasing number of co-morbidities [12]. Specific chronic diseases that have been associated with falling are Chronic Obstructive Pulmonary Disease (COPD), cardiovascular disease, diabetes, previous stroke, thyroid disease and arthritis [2,12,13,16], along with mental illnesses such as depression and cognitive impairment [12,15]. Other diseases considered in this model are asthma, any cancer, epilepsy, multiple sclerosis, osteoporosis, and urinary tract infection.

There are strong associations between patient medications and falls risk. Specific drugs with a firm evidence base are benzodiazepines [13,19–21], psychotropic drugs [12,14,20–22], antidepressants [12,13,19], and some cardiovascular medications including digoxin, class 1A anti-arrhythmic drugs, ACE-inhibitors, and diuretics [12,23]. Further to these specific drugs, polypharmacy (usually defined as 4 or more regular medications) has also been frequently implicated as a risk factor for falls [12,13,16,23,24]. In addition, statins have also been considered in the model due to their association with proximal myopathy and therefore leg weakness.

### Definition of Primary Outcome

The primary outcome measure for this study was the occurrence of a fall or a fracture or both during the study period. This was determined by the presence of codes for these outcomes in the general practice or hospital data. A full list of codes selected for both falls and fractures can be found in S1 and S2 Tables. Fractures were used as a surrogate marker for falls where a fall itself was not documented, as over 90% of fractures occur as a result of a fall [1].

### Cost Analysis

To undertake our cost analysis, the cost of a fall, cost of a falls intervention, and the efficacy of falls interventions were required. The cost of a fall was taken as the difference between the health utilisation, calculated using the Payment by Results methodology [25], for those that fell and those that did not, in the year following the index date. Other values for the cost of a fall were also considered, and the results of these can be found in S4 Table.

The cost of a falls intervention was taken as the mean cost from the literature of multifactorial falls interventions [26–28], as this is the intervention recommended by The National Institute for Health and Care Excellence (NICE) [29]. A recent Cochrane review found multifactorial falls interventions to produce a relative rate reduction of 24% [30], therefore this value was used to represent the efficacy of a falls intervention.

### Statistical Analysis

Multilevel logistic regression analysis was performed to identify predictors of falls. The strongest predictors were used to generate a risk model. Multilevel analysis was performed in order to adjust for variation between populations based on general practice. A two level model was used with patients nested with their general practice. The random effect was the general practice, which may reflect quality of care but might be a proxy for other factors, such as deprivation and other characteristics of the locality not included in the model. For each of the variables, the strength of the association and impact on the Bayesian information criterion value was calculated, and variables without a statistically significant association (p<0.05) were removed from the final risk model. Odds ratios (OR) with 95% confidence intervals were calculated for each of the variables included in the final risk profile model (Table 1).

**Table 1:** Risk profile model for occurrence of falls, obtained by multi-level logistical regression.

Predictor	Regression Coefficient	Odds ratio	95% CI	*p* Value
**Age 65–69**	Reference	-	-	-
**Age 70–74**	0.41	1.51	1.32, 1.73	<0.001
**Age 75–79**	1.18	3.24	2.86, 3.67	<0.001
**Age 80–84**	1.52	4.57	4.03, 5.17	<0.001
**Age 85–89**	2.00	7.37	6.46, 8.42	<0.001
**Age 90–94**	2.00	7.40	6.33, 8.66	<0.001
**Age 95+**	0.77	2.17	1.75, 2.68	<0.001
**Female Sex**	0.12	1.13	1.06, 1.21	<0.001
**Inpatient episode previous 1 month**	0.86	2.37	2.00, 2.81	<0.001
**Inpatient episode previous 2 months**	-0.21	0.81	0.69, 0.95	<0.01
**1 outpatient visit previous 1 month**	0.13	1.14	1.03, 1.26	<0.05
**1–5 outpatient visits previous 12 months**	0.22	1.25	1.16, 1.35	<0.001
**6–10 outpatient visits previous 12 months**	0.32	1.38	1.21, 1.57	<0.001
**≥11 outpatient visits previous 12 months**	0.36	1.43	1.20, 1.69	<0.001
**A+E investigation previous 3 months**	-0.15	0.86	0.74, 0.99	<0.05
**Non-elective admission in previous year**	0.32	1.38	1.24, 1.54	<0.001
**GP code of a fracture (>6 months ago)**	0.32	1.38	1.23, 1.54	<0.001
**GP code of a fracture (previous 6 months)**	0.95	2.58	2.04, 3.26	<0.001
**Osteoporosis**	0.31	1.36	1.17, 1.60	<0.001
**GP/Hospital Code of fall (>6 months ago)**	0.50	1.65	1.35, 2.01	<0.001
**GP/Hospital code of fall (previous 6 months)**	0.93	2.53	2.13, 3.01	<0.001
**COPD (Long term condition)**	0.20	1.22	1.09, 1.37	<0.001
**History of stroke**	0.16	1.18	1.06, 1.31	<0.01
**Depression (Long term condition)**	0.28	1.33	1.20, 1.47	<0.001
**Mental Health (Long term condition)**	0.39	1.48	1.20, 1.83	<0.001
**Asthma (Long term condition)**	0.30	1.34	1.13, 1.60	<0.01
**History of urinary tract infection**	0.94	2.56	2.32, 2.81	<0.001
**Polypharmacy 1–4 unique drugs**	0.88	2.41	2.12, 2.73	<0.001
**Polypharmacy 5–9 unique drugs**	1.00	2.71	2.40, 3.06	<0.001
**Polypharmacy ≥10 unique drugs**	1.02	2.76	2.42, 3.15	<0.001

A full list of variables considered can be found in S3 Table.

The predictive accuracy of the model was expressed using the area under the curve (AUC) of the receiver operating characteristics (ROC) curve, sensitivity and specificity, and Youden’s index (sensitivity + specificity—1). Further validation studies were also carried out, including generating a Hosmer-Lemeshow plot for goodness-of-fit across different sub-populations, and Variance Inflation Factor (VIF), to assess for co-linearity. All statistical tests were carried out using R (Version 3.1.1), and the ‘lme4’ package was used to produce the model. “lme4” can be obtained from the Comprehensive R Archive Network (CRAN, https://cran.r-project.org/web/packages/lme4/index.html).

For the cost analysis, a Monte Carlo Model (MCM) simulation was used to generate a distribution around the key variables for the cost analysis; the cost of a fall, the cost of a falls intervention, and the effectiveness of a falls intervention [31]. The MCM simulation allowed for us to adjust for uncertainty in the estimates of these variables, by sampling from this distribution. A matrix was produced containing 100,000 values within 95% confidence intervals around the key variables for the cost analysis. Savings were calculated as the cost of the falls intervention in the intervened population minus the cost of the potential falls that were prevented in the intervened group. Using the matrix generated by MCM simulation, we produced the mean and confidence range, of the net savings with cut-off risk values at intervals of 0.01.

### Ethical Considerations

This study formed part of the evaluation of the ICP. The data used were those recorded for routine care. This evaluation was retrospective and did not impact on any healthcare decisions made between doctor and patient. The data used were anonymised and no patient identifiable information was used. This investigation was approved by the ICP steering group.

## Results

### Study Population

Data was collected on 74751 individuals over a 2.5 year period. During the follow up period 4941 subjects (6.6%) presented to their GP or to hospital with either a fall or a fracture or both. There were 40249 females (54%) and 34502 males (46%) in the study population. This greater proportion of females is reflective of the general elderly population and likely to reflect differences in life expectancy between genders [32]. Prevalence of falls was greater in females (8.3%) compared to males (5.2%). All of the population were aged 65 and over. The distribution of subjects among the age groups was: 21700 in 65–69, 16101 in 70–74, 12469 in 75–79, 8972 in 80–84, 5323 in 85–89, 2522 in 90–94, and 7664 in 95+.

### Risk Model

**Fig 1:**
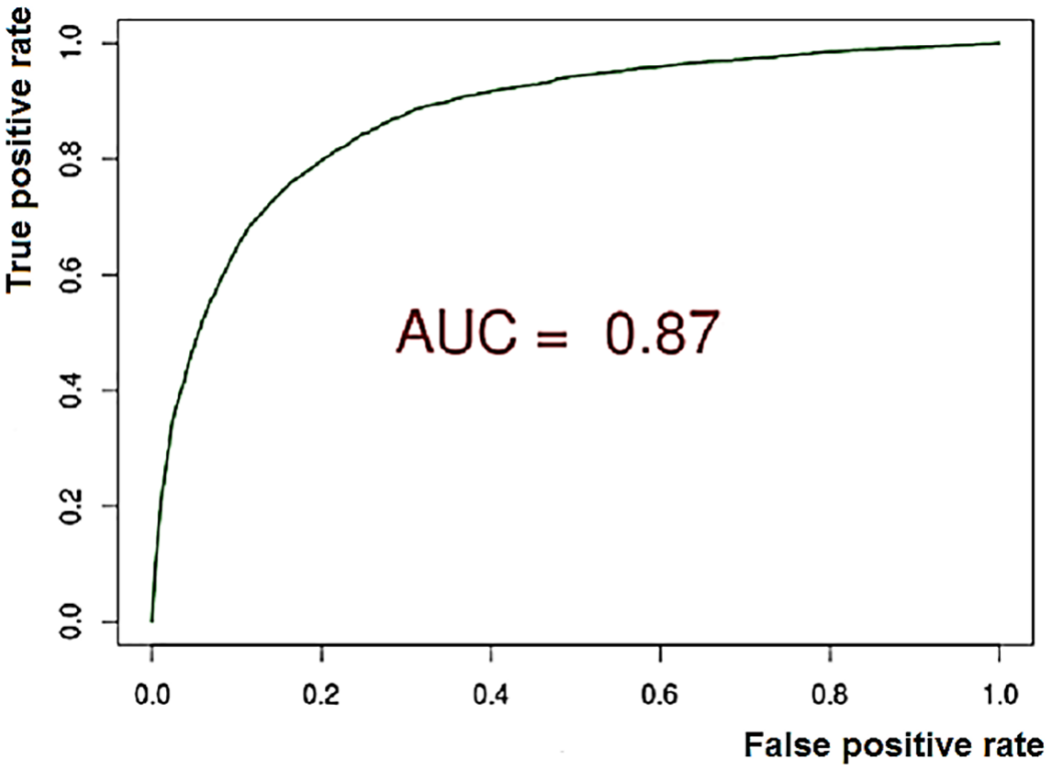
Receiver operating characteristics curve of the falls risk prediction model. AUC = 0.87.

Multilevel logistic regression analysis was performed to identify predictors of falls. The strongest predictors were used to generate a risk model, and variables without a statistically significant association (p<0.05) were removed from the final risk model in a stepwise fashion. Tests of validity were carried out on the risk model. A Hosmer-Lemeshow plot, for goodness-of-fit, indicated that the model was well calibrated. The ROC curve demonstrated a high level of predictive accuracy, with an area under the curve of 0.87 (Fig 1).

Tests for co-linearity were also carried out, which confirmed that there was no significant correlation between any variables in the model, with no variables having a Variance Inflation Factor greater than 3. Female sex, increasing age, an inpatient episode in the previous 1 month, outpatient visits within the previous 1 and 12 months, polypharmacy, asthma, COPD, depression, mental illness, previous stroke, previous urinary tract infection, non-elective hospital admission in the previous 12 months, GP coding of a previous fracture, osteoporosis, and a previous fall were all identified as significant risk factors contributing to our risk model (Table 1). The level of risk (Odds Ratio) increased with increasing age, with the exception of the over 95 age group. The level of risk also increased with increasing number of outpatient visits in the previous 12 months, and with increasing number of drugs (polypharmacy).

Further to this, risk was higher for a fracture or a fall occurring in the previous 6 months, as opposed to any past history of a fracture or a fall. An inpatient episode in the previous 2 months, and an A&E investigation in the previous 3 months, were both protective for falls. A total of 116 variables were considered in construction of the risk model. A full list of variables that were excluded due to lack of a significant association with a fall or fracture can be found in S3 Table. Notable variables excluded from the model, that based on existing evidence would have been expected to be risk factors, included: deprivation (Index of multiple deprivation score), disability [14] and vulnerability.

Chronic disease in itself [12] was also excluded, along with and specific chronic diseases such as cardiovascular disease, diabetes, thyroid disease and arthritis, cognitive impairment, any cancer, epilepsy and multiple sclerosis [12,15]. Although polypharmacy was a significant risk factor in our model, specific drugs with very strong evidence as falls risk factors, such as psychotropic drugs [12,14,20–22] including benzodiazepines[13,19–21] and antidepressants[12,13,19], and cardiovascular medications including statins, digoxin, class 1A anti-arrhythmic drugs, ACE-inhibitors, and diuretics [12,23] were all excluded from the model due to lack of a significant association.

**Fig 2:**
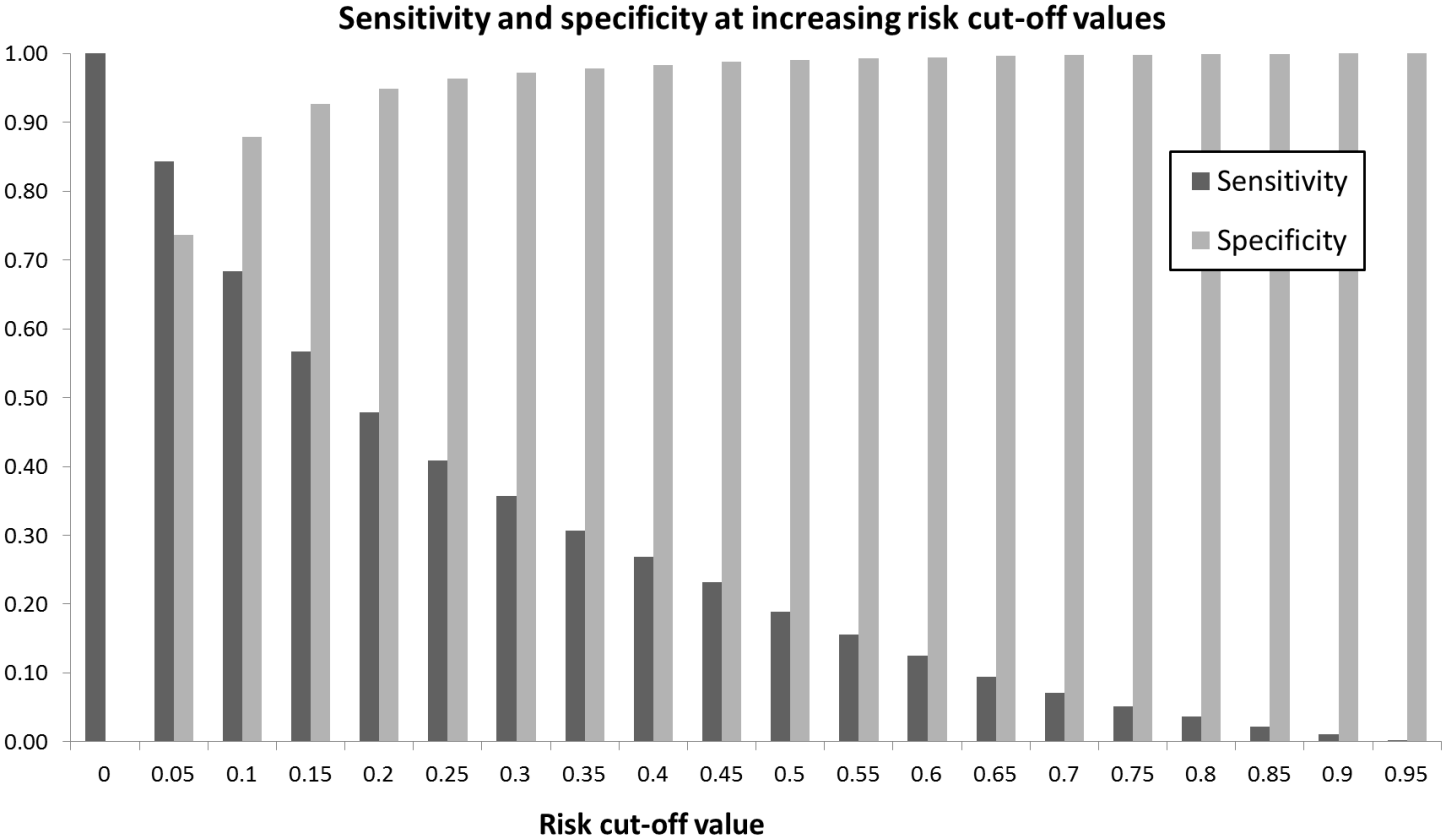
Sensitivity and specificity of our risk model for cut off values at intervals of 0.05. The cut-off with the highest combination of sensitivity and specificity was a risk cut-off value of 0.07.

To consider our risk model as a predictive tool, diagnostic and predictive values using risk cut-off values were calculated for intervals of 0.01, and of 0.05 (Table 2). The cut-off with the highest combination of sensitivity and specificity was a risk cut-off value of 0.07 (Fig 2). This cut-off produced a sensitivity of 81% and specificity of 78%. The Negative predictive value at this cut-off was 98% and positive predictive value was 21%.

**Table 2:** Table showing cost calculations for varying risk level cut off values.

Risk cut-off value	% referred	% of referred that fell	% of all falls in referred	Net cost/savings	95% Confidence range
**0**	100.00%	3.27%	100.00%	-£40,556,126	(-£56,203,851,-£24,918,962)
**0.05**	12.97%	16.02%	63.62%	-£3,328,324	(-£5,389,709,-£1,267,138)
**0.1**	6.44%	23.07%	45.47%	-£1,113,385	(-£2,156,610,-£61,919)
**0.15**	4.11%	28.29%	35.60%	-£456,673	(-£1,134,394,£229,348)
**0.2**	2.87%	32.79%	28.84%	-£166,137	(-£651,229,£327,255)
**0.25**	2.09%	36.93%	23.68%	-£18,320	(-£380,704,£351,620)
**0.3**	1.54%	40.55%	19.17%	£52,217	(-£222,549,£330,992)
**0.35**	1.17%	43.43%	15.57%	£78,783	(-£133,869,£297,243)
**0.4**	0.93%	45.10%	12.82%	£81,708	(-£90,506,£258,383)
**0.45**	0.70%	48.47%	10.36%	£89,246	(-£45,049,£228,022)
**0.5**	0.53%	50.89%	8.23%	£82,664	(-£21,896,£193,038)
**0.55**	0.37%	58.27%	6.64%	£90,561	(£11,294,£177,565)
**0.6**	0.26%	62.69%	4.96%	£76,936	(£17,834,£142,795)
**0.65**	0.18%	66.92%	3.65%	£61,092	(£16,998,£112,168)
**0.7**	0.11%	71.60%	2.38%	£43,017	(£12,747,£81,044)
**0.75**	0.06%	86.05%	1.52%	£32,594	(£11,869,£61,318)
**0.8**	0.03%	90.48%	0.78%	£17,130	(£4,394,£36,510)
**0.85**	0.01%	90.00%	0.37%	£7,901	(£224,£20,732)

Each cut off is the risk value above which subjects would be referred to a falls intervention.

### Cost Analysis

**Fig 3:**
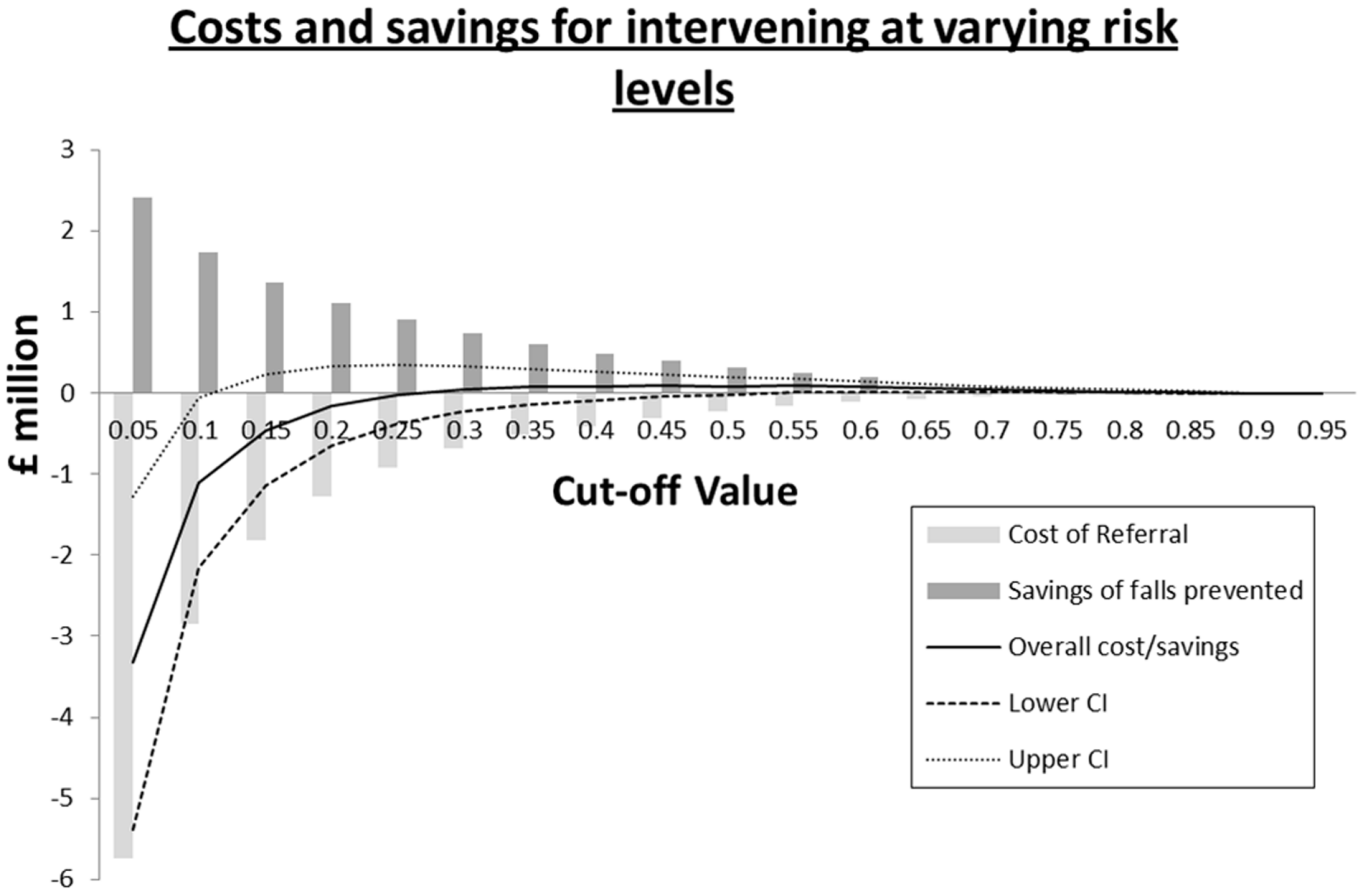
A graph demonstrating the potential savings (or costs) to be made by intervening at varying levels of risk. The mean (solid line), upper (dotted line) and lower (dashed line) 95% confidence intervals for net savings are represented along with bars for the cost of referral and savings from prevented falls.

If no cut-off value is used, and 100% of the population is sent to a falls intervention, at a cost of £592 per patient, the total cost of intervening in our study population would be £44.2 million. 585 falls would be prevented, producing a saving of £3.6 million. This results in an overall cost of £40.6 million (95% Confidence Interval £24.9 million, £56.2 million) (Table 2). As the cut-off value is increased, the trend of costs exceeding savings continues until a cut-off risk value of p = 0.27. At this value, 1376 individuals (1.8% of the population) would be sent to the falls intervention, at a cost of £0.81 million. 127 falls would be prevented, producing a saving of £0.83 million. This would result in an overall net saving of £15889 (95% CI -£306289, £344415) (Fig 3). As the cut-off value increases further, net savings continue to increase, to a maximum of £92810 (95% CI £2677, £189932), at p = 0.53. Beyond a cut-off of p = 0.53 net savings begin to fall, however never below 0.

## Discussion

### Principal Findings

Our model performs better than any other existing model or assessment tool to assess risk of falling. The AUC of the ROC curve is 0.87. The maximum combination of specificity and sensitivity is achieved at a risk cut-off of ≥0.07. At this cut-off, sensitivity is 0.78 and specificity is 0.81, creating a Youden’s index of 0.59, positive predictive value (PPV) is 0.23 and negative predictive value (NPV) is 0.98.

The next nearest contender, a prospective cohort study by Stalenhoef et al gathered data over a 36 week period and produced a risk model with an AUC of the ROC curve of 0.79 [33]. This risk model was produced from data on 311 patients, versus the 74751 participants in our study. Our model runs on routine data from primary and secondary care and performs better than our previous falls risk model produced from primary care data alone [8]. Of the final 29 variables forming our risk model, 10 of these are variables obtained from hospital data, and endpoints were collated from both general practice and hospital data. Our previous falls model produced a maximum sensitivity and specificity at a cut-off score of 9, sensitivity was 0.68 and specificity was 0.60, with an AUC of the ROC curve of 0.72, versus the 0.78, 0.81 and 0.87 respectively of the model presented in this paper.

Our falls risk model can be applied in a way that saves health services costs. Cost analysis was successfully applied to elucidate those patients in whom cost savings could be made through referral to a falls prevention service. At low cut-offs, there are considerably large net costs, because the costs of referring a large proportion of the population to a falls prevention service outweigh the savings made by preventing relatively few falls (Fig 2). At a cut-off value of p = 0.27, the savings from falls prevented exceed the costs of referral. At this cut off 1.8% of the population would be referred and the prevalence of falls among this population is 38%. Net savings increase as the cut-off increases beyond 0.27, to a peak at p = 0.53. Beyond 0.53, net savings fall, but never back below 0, into net costs. This means that above a risk score of 0.27, savings can be made by referral to a falls prevention service.

### Implications of findings

In optimum use, this model will identify the top 22% most at risk of falling within a population with a sensitivity of 0.78, a specificity of 0.81, PPV of 0.23 and NPV of 0.98 at a cut-off value of ≥0.07. Based on our data, this 22% of the population would include 77% of all falls. Due to our model being produced from routine healthcare data, it could be implemented across healthcare services within the UK or adapted to run in systems with similar health data. We have provided the full list of variables considered for the model and code lists used to produce these variables in the supporting documents.

Our model could be integrated into a general practice or integrated care database to identify high risk patients at an individual population level. Through use of the findings of our cost-analysis, users could identify those patients in whom savings could be made through referral to a falls prevention service. Further savings can be attributed to the very minimal screening time and costs compared to conventional screening methods which cost approximately £165 per patient [26].

We recommend that a cut-off value of 0.27 is used to guide referral to falls prevention services: the value at which savings from prevented falls outweigh the costs of referral. At this value the most at risk 1.8% of the over 65 population would be referred. In a moderate sized general practice of 10,000 patients this would result in referral of 31 individuals [32]. Service providers may however prefer to use a cut-off that produces the maximum level of savings, which would be achieved at a higher cut-off risk value of 0.53. This would result in referral of the most at risk 0.45% of the over 65 population (8 individuals from a population of 10,000).

### Comparison to the literature

A literature review of existing falls risk models and tools (S5 Table), was unable to yield any models with superior predictive capabilities (AUC of the ROC curve) to our risk model. The most frequently used tool in falls services in the UK is the Falls Risk Assessment Tool (FRAT) [6]. A study of the FRAT did not provide the AUC, but instead presented Youden’s Index as a predictive measure. The maximum Youden’s Index was 0.39, for a cut-off of ≥2 [5], compared to 0.56 at a cut-off of 0.07 in our model. In addition to the superior predictive capabilities of our model, entire populations could be screened in minutes, versus a time consuming individual assessment required in other tools such as FRAT.

While several studies have investigated the cost-effectiveness of multifactorial falls interventions, in the UK [26] and abroad [27,34–36], no studies (of any type of falls intervention) could be found that apply cost-analysis at varying levels of risk, or that aim to identify those in whom the intervention would be cost effective, as has been carried out in our research; although one study did identify that an intervention was more cost effective in the higher risk population [37]. The majority of these studies show multifactorial interventions to not be cost effective. However, as highlighted by our research, this does not mean that they would not be cost effective, if the correct population were referred.

### Limitations

Although also a clear strength, one limitation of this study is that the database was derived from routinely collected general practice and hospital data. The general practice data was derived from Read Codes recorded during consultations and is therefore reliant on accurate code selection and on hospital attendances with falls/fractures being communicated. Hospital data was derived from ICD-10 codes recorded by hospital coders and is therefore reliant on accurate code selection and on the hospital doctors having documented that a fall/fracture had occurred. We have minimised this effect by combining falls from both general practice and hospital data in the analysis. Not all elderly people who fall will go to their general practice or to the hospital. Medical attention will likely only be sought if there are multiple falls or the mechanism of the fall or the resultant injuries are significant enough to require medical assistance. A Cochrane review has shown that only 20% of falls result in injury significant enough to require medical attention [30]. This means that a significant proportion of falls will not be included in the data, which is of particular importance given that previous falls have consistently been shown to be a predictor of subsequent falls [4]. A potential benefit of minor falls not being represented is that the falling population included within our risk model are those who have required medical attention resulting from a fall, and are therefore likely to represent a higher risk population that would benefit more from intervention. A further, unavoidable limitation of the study is that the inclusion criteria stipulated that subjects must be registered with their general practice for the entire study period. This unfortunately excludes those subjects that died during the study period. As such, any falls occurring in this population have not been included in the analysis. Our MCM simulation assumed that the various cost and benefits of a fall were uncorrelated, e.g. there was no correlation between the cost of an intervention and its chance of success. This assumption may be unrealistic; however there is no prior literature that could be used to inform this model. Further limitations are that the model was developed with data from a single area, albeit with a population of 656902, and it has not been validated on an external population.

### Further research

Further work is needed to validate this risk model in other populations. A randomised controlled trial also needs to be performed, in which the risk model is applied to a general practice population to identify high risk individuals for referral to a falls prevention service, to elucidate whether the application of the model can firstly reduce the number of falls or fractures occurring in this population, and secondly produce savings through the prevention of these falls.

The model in its current iteration is produced from integrated care data. Since integrated care is currently a limited concept, most general practices are likely to have access to only general practice records. Therefore a version of the risk model with general practice only variables will need to be produced. This will improve the ease of use and widen its application possibilities. An Excel spreadsheet illustrating the model has been produced and is available.

## Conclusion

This model is the best performing falls predictive tool developed to date; it has been developed on a large UK city population; can be readily run from routine data; and can be implemented in a way that optimises the use of health service resources. Its limitations are that it was developed in a single area, albeit with a population of 656,902; it has not been tested in a trial; and our algorithm is only available using the statistical programme R. Commissioners of health services should use this model to flag and refer patients at risk to their falls service and save resources.

## Supporting Information

S1 File Appendix 6 –Supplementary references. (DOCX)

S1 Table Appendix 1a –List of codes used to produce disease variables. (DOCX)

S2 Table Appendix 1b- List of codes used to produce drug variables. (DOCX)

S3 Table Appendix 2 –List of variables. (DOCX)

S4 Table Appendix 3- Alternative cost analysis. (DOCX)

S5 Table Appendix 4 –Comparison of risk model with existing models. (DOCX)

S6 Table Appendix 5 –STROBE statement. (DOCX)
