# Supplementary material for: Predicting Falls and When to Intervene in Older People: A Multilevel Logistical Regression Model and Cost Analysis
Source: PLoS One. 2016 Jul 22;11(7):e0159365. doi: 10.1371/journal.pone.0159365 (PMC4957756; doi:10.1371/journal.pone.0159365)
Supplement: S1 File — (DOCX) [file pone.0159365.s001.docx]

**Appendix 6 – Supplementary references**

38. Tian Y, Thompson J, Buck D, Sonola L. Exploring the system-wide costs of falls in older people in Torbay. London: The King’s Fund [Internet]. 2013 [cited 2014 Aug 14]; Available from: http://www.kingsfund.org.uk/sites/files/kf/field/field_publication_file/exploring-system-wide-costs-of-falls-in-torbay-kingsfund-aug13.pdf

39. Scuffham P, Chaplin S, Legood R. Incidence and costs of unintentional falls in older people in the United Kingdom. 2003;(table 1):70–4. Available from: http://jech.bmj.com/content/57/9/740.full.pdf+html

40. Demura S, Sato S, Shin S, Uchiyama M. Setting the criterion for fall risk screening for healthy community-dwelling elderly. Archives of gerontology and geriatrics [Internet]. Elsevier Ireland Ltd; 2012 [cited 2014 Oct 21];54(2):370–3. Available from: http://www.ncbi.nlm.nih.gov/pubmed/21570727

41. Woo J, Leung J, Wong S, Kwok T, Lee J, Lynn H. Development of a simple scoring tool in the primary care setting for prediction of recurrent falls in men and women aged 65 years and over living in the community. Journal of clinical nursing [Internet]. 2009 Apr [cited 2014 Oct 2];18(7):1038–48. Available from: http://www.ncbi.nlm.nih.gov/pubmed/19207793

42. Stalenhoef P a, Diederiks JP, Knottnerus J a, De Witte LP, Crebolder HF. The construction of a patient record-based risk model for recurrent falls among elderly people living in the community. Family practice [Internet]. 2000 Dec;17(6):490–6. Available from: http://www.ncbi.nlm.nih.gov/pubmed/11120721

43. Russell M a, Hill KD, Day LM, Blackberry I, Gurrin LC, Dharmage SC. Development of the Falls Risk for Older People in the Community (FROP-Com) screening tool. Age and ageing [Internet]. 2009 Jan [cited 2014 Oct 22];38(1):40–6. Available from: http://www.ncbi.nlm.nih.gov/pubmed/19141507

44. Gunn H, Creanor S, Haas B, Marsden J, Freeman J. Risk factors for falls in multiple sclerosis: an observational study. Multiple sclerosis (Houndmills, Basingstoke, England) [Internet]. 2013 Dec [cited 2014 Oct 24];19(14):1913–22. Available from: http://www.ncbi.nlm.nih.gov/pubmed/23633067

45. Tromp AM, Pluijm SMF, Smit JH, Deeg DJH, Bouter LM, Lips P. Fall-risk screening test : A prospective study on predictors for falls in community-dwelling elderly. 2001;54:837–44.

46. Pluijm SMF, Smit JH, Tromp E a M, Stel VS, Deeg DJH, Bouter LM, et al. A risk profile for identifying community-dwelling elderly with a high risk of recurrent falling: results of a 3-year prospective study. Osteoporosis international : a journal established as result of cooperation between the European Foundation for Osteoporosis and the National Osteoporosis Foundation of the USA [Internet]. 2006 Jan [cited 2014 Oct 22];17(3):417–25. Available from: http://www.ncbi.nlm.nih.gov/pubmed/16416256

47. Close J, Ellis M, Hooper R, Glucksman E, Jackson S, Swift C. Prevention of falls in the elderly trial (PROFET): a randomised controlled trial. Lancet [Internet]. 1999 Jan 9;353(9147):93–7. Available from: http://www.ncbi.nlm.nih.gov/pubmed/10023893

48. Bongue B, Dupré C, Beauchet O, Rossat A, Fantino B, Colvez A. A screening tool with five risk factors was developed for fall-risk prediction in community-dwelling elderly. Journal of clinical epidemiology [Internet]. 2011 Oct [cited 2014 Oct 14];64(10):1152–60. Available from: http://www.ncbi.nlm.nih.gov/pubmed/21463927

49. Tiedemann A, Sherrington C, Orr T, Hallen J, Lewis D, Kelly A, et al. Identifying older people at high risk of future falls: development and validation of a screening tool for use in emergency departments. Emergency medicine journal : EMJ [Internet]. 2013 Nov [cited 2014 Oct 20];30(11):918–22. Available from: http://www.ncbi.nlm.nih.gov/pubmed/23139096

50. Hohtari-Kivimäki U, Salminen M, Vahlberg T, Kivelä S-L. Short Berg Balance Scale, BBS-9, as a predictor of fall risk among the aged: a prospective 12-month follow-up study. Aging clinical and experimental research [Internet]. 2013 Dec [cited 2014 Oct 24];25(6):645–50. Available from: http://www.ncbi.nlm.nih.gov/pubmed/24170328

51. Lin M-R, Hwang H-F, Hu M-H, Wu H-DI, Wang Y-W, Huang F-C. Psychometric comparisons of the timed up and go, one-leg stand, functional reach, and Tinetti balance measures in community-dwelling older people. Journal of the American Geriatrics Society [Internet]. 2004 Aug;52(8):1343–8. Available from: http://www.ncbi.nlm.nih.gov/pubmed/15271124

52. Barry E, Galvin R, Keogh C, Horgan F, Fahey T. Is the Timed Up and Go test a useful predictor of risk of falls in community dwelling older adults: a systematic review and meta- analysis. BMC geriatrics [Internet]. BMC Geriatrics; 2014 Jan [cited 2014 Oct 9];14(1):14. Available from: http://www.pubmedcentral.nih.gov/articlerender.fcgi?artid=3924230&tool=pmcentrez&rendertype=abstract

53. Oliver D, Britton M, Seed P, Martin FC, Hopper AH. Development and evaluation of evidence based risk assessment tool (STRATIFY) to predict which elderly inpatients will fall: case-control and cohort studies. BMJ [Internet]. 1997 Oct 25 [cited 2014 Sep 12];315(7115):1049–53. Available from: http://www.bmj.com/cgi/doi/10.1136/bmj.315.7115.1049

54. Hendrich AL, Bender PS, Nyhuis A. Validation of the Hendrich II Fall Risk Model: a large concurrent case/control study of hospitalized patients. Applied nursing research : ANR [Internet]. 2003 Feb [cited 2014 Sep 12];16(1):9–21. Available from: http://www.ncbi.nlm.nih.gov/pubmed/12624858
