# Supplementary material for: Predicting Falls and When to Intervene in Older People: A Multilevel Logistical Regression Model and Cost Analysis
Source: PLoS One. 2016 Jul 22;11(7):e0159365. doi: 10.1371/journal.pone.0159365 (PMC4957756; doi:10.1371/journal.pone.0159365)
Supplement: S1 Table — (DOCX) [file pone.0159365.s002.docx]

**Appendix 1a – List of codes used to produce disease variables**

| **Falls in hospital data** |  |  |  |  |
| --- | --- | --- | --- | --- |
| **Descriptor** | **ICD-10** | **SNOMED-CT** | **Read Code** | **SNOMED-RT** |
| Tendency to fall, not elsewhere classified | R29.6 | N/A | N/A | N/A |
| Fall on same level involving ice and snow | W00 | 435521000000108(UK) | U100 | A-A1801 |
| Fall on same level from slipping, tripping and stumbling | W01 | 407281000000106(UK) | U101 | A-A1885 |
| Other fall on same level due to collision with, or pushing by, another person | W03 | 436311000000103(UK) | U103 | A-A182A |
| Fall while being carried or supported by other persons | W04 | 388911000000103(UK) | U104 | A-A1839 |
| Fall involving wheelchair | W05 | 17886000 | U105 | A-A1885 |
| Fall involving bed | W06 | 20902002 | U106 | A-A1882 |
| Fall involving chair | W07 | 83468000 | U107 | A-A1884 |
| Fall involving other furniture | W08 | 448321000000106(UK) | U108 | A-A1846 |
| Fall on and from stairs and steps | W10 | 217083007/395341000000105 (UK) | TC0../U10A | A-A1F61 |
| Other fall from one level to another | W17 | 217146009/217139000 | TC4y./ TC4.. | A-A16DA/ R-F045D |
| Other fall on same level | W18 | 33036003 | XUBt7 | A-A1802 |
| Unspecified fall | W19 | 394781000000102 (UK) | U10z | A-A18DE |
|  |  |  |  |  |
| **Falls in GP data** |  |  |  |  |
| **Descriptor** | **ICD-10** | **SNOMED-CT** | **Read Code** | **SNOMED-RT** |
| Accidental falls | N/A | 217082002 | TC.. | A-A1F60 |
| Falls | N/A | 1912002 | U10.. | A-A1800 |
| Number of falls in last year (where ‘value’ field shows a value greater than zero) | N/A | 391002003 | 16D../XaISO | F-02B8C |
|  |  |  |  |  |
|  |  |  |  |  |
| **Osteoporotic fracture** |  |  |  |  |
| **Descriptor** | **ICD-10** | **SNOMED-CT** | **Read Code** | **SNOMED-RT** |
| Postmenopausal osteoporosis with pathological fracture | M80.0 | 203453001 | N331B | DD-10056 |
| Postoophorectomy osteoporosis with pathological fracture | M80.1 | 203444008 | N3312 | D1-61516 |
| Osteoporosis of disuse with a pathological fracture | M80.2 | 203445009 | N3313 | DD-10052 |
| Postsurgical malabsorption osteoporosis with pathological fracture | M80.3 | 203446005 | N3314 | DD-10053 |
| Drug-induced osteoporosis with pathological fracture | M80.4 | 203447001 | N3315 | DD-10054 |
| Idiopathic osteoporosis with pathological fracture | M80.5 | 203448006 | N3316 | DD-10055 |
| Other osteoporosis with pathological fracture | M80.8 | 439581000000106 (UK) | NyuB0 | D1-6150A |
| Unspecified osteoporosis with pathological fracture | M80.9 | 425371000000103(UK) | NyuB8 | D1-6150D |
|  |  |  |  |  |
| **Fracture of spine** |  |  |  |  |
| **Descriptor** | **ICD-10** | **SNOMED-CT** | **Read Code** | **SNOMED-RT** |
| Fatigue fracture of vertebra | M48.4 | 202831001 | N1y1 | DD-11028 |
| Collapsed vertebra, not elsewhere classified | M48.5 | 390801000000109(UK) | Nyu67 | DD-11024 |
| Fracture of thoracic vertebra | S22.0 | (Fracture of thoracic spine) 125607007 | XA0GM | DD-1103B |
| Multiple fractures of thoracic spine | S22.1 | 208215006 | S150 | DD-110B2 |
| Fracture of lumbar vertebra | S32.0 | (Fracture of Lumbar spine) 125608002 | XA0GN | DD-1103C |
| Fracture of sacrum | S32.1 | 125872003 | XA0GO | DD-1103D |
| Fracture of coccyx | S32.2 | 125871005 | XA0GP | DD-1103E |
| Fracture of spine, level unspecified | T08.X | N/A | N/A | N/A |
|  |  |  |  |  |
| **Fracture of pelvis** |  |  |  |  |
| **Descriptor** | **ICD-10** | **SNOMED-CT** | **Read Code** | **SNOMED-RT** |
| Fracture of ilium | S32.3 | 7687006 | XA0H2 | DD-11519 |
| Fracture of acetabulum | S32.4 | 64455005 | XA0H1 | DD-11513 |
| Multiple fractures of lumbar spine and pelvis | S32.7 | 207993005 | S10B6 | DD-100CF |
|  |  |  |  |  |
| **Fracture of arm/wrist** |  |  |  |  |
| **Descriptor** | **ICD-10** | **SNOMED-CT** | **Read Code** | **SNOMED-RT** |
| Fracture of upper end of humerus | S42.2 | 127286005] | XA0GQ | DD-12211 |
| Fracture of shaft of humerus | S42.3 | 50890004 | XA0GS | DD-12230 |
| Fracture of lower end of humerus | S42.4 | 263192005 | XA0GT | DD-12206 |
| Fracture of upper end of ulna | S52.0 | 263203001 | XA0Gi | DD-12317 |
| Fracture of upper end of radius | S52.1 | 263195007 | XA0GX | DD-1230E |
| Fracture of shaft of ulna | S52.2 | 263204007 | XA0Gj | DD-12318 |
| Fracture of shaft of radius | S52.3 | 263198009 | XA0Ga | DD-12313 |
| Fracture of shafts of both ulna and radius | S52.4 | 75857000 | XUKQA | DD-12306 |
| Fracture of lower end of radius | S52.5 | 263199001 | XA0Gb | DD-12315 |
| Fracture of lower end of both ulna and radius | S52.6 | 263208005 | XA0Gn | DD-1231C |
| Multiple fractures of forearm | S52.7 | 208513000 | S293 | DD-1232E |
| Fracture other parts of forearm | S52.8 | 479721000000102(UK) | Syu53 | DD-12301 |
| Fracture of forearm, part unspecified | S52.9 | 208293003 | S2300 | DD-1233B |
| Fracture of navicular [scaphoid] bone of hand | S62.0 | 31975004 | XA0Gp | DD-12401 |
|  |  |  |  |  |
| **Fracture of hip** |  |  |  |  |
| **Descriptor** | **ICD-10** | **SNOMED-CT** | **Read Code** | **SNOMED-RT** |
| Fracture of neck of femur | S72.0 | 5913000 | XE1l3 | DD-13110 |
| Pertrochanteric fracture | S72.1 | 208562000 | S304 | DD-1316E |
| Subtrochanteric fracture | S72.2 | 263229001 | XA0HJ | DD-13109 |
|  |  |  |  |  |
| **Fracture of femur** |  |  |  |  |
| **Descriptor** | **ICD-10** | **SNOMED-CT** | **Read Code** | **SNOMED-RT** |
| Fracture of shaft of femur | S72.3 | 54441004 | XA0HN | DD-13150 |
| Fracture lower end of femur | S72.4 | 263232003 | XA0HO | DD-1310A |
| Fracture of other parts of femur | S72.8 | 480321000000103(UK) | Syu72 | DD-13106 |
| Fracture of femur, part unspecified | S72.9 | 71620000 | XA0HC | DD-13100 |
|  |  |  |  |  |
| **Fragility fractures from GP data** |  |  |  |  |
| **Descriptor** | **ICD-10** | **SNOMED-CT** | **Read Code** | **SNOMED-RT** |
| Closed reduction of fracture of hip | N/A | 179159005 | 7K1L4 | P1-18E1D |
| Fracture therapy follow-up (finding) | N/A | 183653007 | 8HB9. | F-0470F |
| Seen in fracture clinic (finding) | N/A | 185191008 | 9N0X. | F-006A7 |
| Pathological fractures | N/A | 203441000 | N331. | N/A |
| Fracture of upper limb | T10X, T022, T024 | 23406007 | S2% | DD-12000 |
| Fracture of lower limb | T025, T12X, T023 | 46866001 | S3% | DD-13000 |
| TC7 | N/A | 217171007 | TC7% | A-A16E7 |
|  |  |  |  |  |
| **Codes for Nocturia** |  |  |  |  |
| **Description** | **ICD-10** | **SNOMED-CT** | **Read Codes** | **SNOMED-RT** |
| History of stress incontinence | N/A | 161794005 | 1593% | G-0316 |
| Bladder: incontinent | R32X | 165232002 | 3940 | F-72058 |
| Double incontinence | R32X/R15X | 78459008 | 16F../X30C5 | F-0A660 |
| Nocturia | N/A | 139394000 | .1A13 | R-F224E |
| Enuresis | F980 | 8009008 | 1A22%/ X008B | D9-16230 |
| Urinary incontinence | N/A | 165232002 | 1A23. | F-72058 |
| Stress incontinence | N39.3 | 22220005 | 1A24. | F-0A650 |
| Urgent desire to urinate | N/A | 75088002 | 1A25. | F-72210 |
| Urge incontinence of urine | N/A | 87557004 | 1A26. | F-0A670 |
| Incontinence care | N/A | 183001000 | 8C14. | P0-00898 |
| Incontinence control | N/A | 183229008/266826009 | 8D71./ XE0kn | P0-00589 |
| Nocturnal bladder warning system | N/A | 271524000 | 8D73./ XE2x8 | A-008C6 |
| Referral to incontinence clinic | N/A | 415274007 | 8HTX./ XaJtx | P0-20854 |
| Stress incontinence | N39.3 | 22220005 | K198./XE0rR | F-0A650 |
| Stress incontinence - female | N/A | 60241006 | K586. | F-72062 |
| Other specified urinary incontinence | N39.4 | 396301000000104(UK) | Kyu5A | F-72061 |
| Incontinence of urine | R32X | 498851000000102(UK) | R083% | R-20F21 |
| Nocturia | R35X | 498911000000108(UK) | R0842 | R-20F29 |
| Urgency of micturition | R39.1 | 499001000000104(UK) | R0862 | R-20F34 |
|  |  |  |  |  |
| **Codes for OA in GP data** |  |  |  |  |
| **Description** | **ICD-10** | **SNOMED-CT** | **Read Codes** | **SNOMED-RT** |
| History of osteoarthritis | N/A | 161568003 | 14G2. | G-0290 |
| Delivery of rehabilitation for osteoarthritis | N/A | 272461000000106(UK) | 7P204 | N/A |
| Generalised osteoarthritis | M15.0 | 201819000 | N050% | D1-20015 |
| Localised primary osteoarthritis | M190 | 201829007 | N051% | D1-2102F |
| Localised secondary osteoarthritis | M192 | 201847001 | N052% | D1-2104D |
| Localised osteoarthritis, unspecified | N/A | 201862004 | N053% | D1-21018 |
| Oligoarticular osteoarthritis, unspecified | M198 | 201874000 | N054% | D1-2000C |
| Osteoarthritis NOS | M199 | 201886002/201917002 | N05z% | R-F881B |
| Osteoarthritis of spine | N/A | 8847002 | N11D% | D1-23300 |
|  |  |  |  |  |
| **Osteoporosis in GP data** |  |  |  |  |
| **Description** | **ICD-10** | **SNOMED-CT** | **Read Codes** | **SNOMED-RT** |
| History of osteoporosis | N/A | 511631000000105 | 14GB. | N/A |
| Osteoporosis | N/A | 64859006 | N330% | D1-61500 |
| Osteoporotic kyphosis | M402 | 203657009 | N3746 | D1-81412 |
| Other osteoporosis | M818 | 426451000000109 | NyuB1 | D1-6150B |
| Osteoporosis in other disorders classified elsewhere | M828A | 463531000000104 | NyuB2 | D1-6150C |
| Forearm DXA scan result osteoporotic | N/A | 390540001/391060008 | 58E4./XaITM | F-1202B |
| Heel DXA scan result osteoporotic | N/A | 390546007/391065003 | 58EA./XaITR | F-12031 |
| Hip DXA scan result osteoporotic | N/A | 390552008/391070005 | 58EG./XaITW | F-12037 |
| Lumbar DXA scan result osteoporotic | N/A | 390558007/391075000 | 58EM./ XaITb | F-163B4 |
| Femoral neck DEXA scan result osteoporotic | N/A | 355541000000105/ 440100002 | 58EV./XUiDZ | F-12050 |
| Osteoporosis treatment started | N/A | 390602006/391020007 | 66a2./XaISg | P0-00736 |
| Osteoporosis treatment changed | N/A | 390604007/391022004 | 66a4./XaISi | P0-00738 |
| Osteoporosis - no treatment | N/A | 390605008/391023009 | 66a5./XaISj | P0-00739 |
| Osteoporosis - dietary advice | N/A | 390606009/391024003 | 66a6./XaISk | P0-0090C |
| Osteoporosis - diet assessment | N/A | 390607000/391025002 | 66a7./XaISl | P0-0057A |
| Osteoporosis - exercise advice | N/A | 390608005/391026001 | 66a8./XaISm | P0-0090D |
| Osteoporosis-falls prevention | N/A | 390609002/391027005 | 66a9./XaISn | P0-0090E |
| Refer osteoporosis specialist | N/A | 390614003/391033001 | 66aE./XaISt | P0-20812 |
| Health education - osteoporosis | N/A | 394010001/394936006 | 679F./XaImg | P0-00607 |
| Refer to osteoporosis clinic | N/A | 121891000000108/412717001 | 8HTS./XaJdn | P0-20841 |
| Osteoporosis - enhanced services administration | N/A | 505151000000106/505141000000108 | 9kj../XaQR9 | N/A |
| Seen in osteoporosis clinic | N/A | 390687002/391037000 | 9N0h./XaISx | F-00F82 |
