# Supplementary material for: Predicting Falls and When to Intervene in Older People: A Multilevel Logistical Regression Model and Cost Analysis
Source: PLoS One. 2016 Jul 22;11(7):e0159365. doi: 10.1371/journal.pone.0159365 (PMC4957756; doi:10.1371/journal.pone.0159365)
Supplement: S2 Table — (DOCX) [file pone.0159365.s003.docx]

**Appendix 1b- List of codes used to produce drug variables**

| **Drugs:** |  |
| --- | --- |
| **Psychotropic drugs, high risk:** |  |
| Sedatives: Benzodiazepines | Read Code |
| Benzodiazepine clinical management plan | 8CR9. |
| Temazepam | d1a% |
| Nitrazepam | d18%, d14% |
| Diazepam | do1%, d21%, j81.. |
| Chlordiazepoxide | d24% |
| Flurazepam | d15% |
| Lorazepam | d2a%, do4%, o56% |
| Oxazepam | d2d.. |
| Clonazepam | dn4%, do2% |
|  |  |
| Sedatives: “Zs” | Read Code |
| Zopiclone | d1d% |
| Zolpidem | d1f% |
|  |  |
| Sedating antidepressants (tricyclics and related drugs) | Read Code |
| Amitriptyline | d71%, gd1.. |
| Dosulepin | d75% |
| Imipramine | d77%, gd5.. |
| Doxepin | d76%, m35% |
| Clomipramine | d73% |
| Lofepramine | d79% |
| Nortriptyline | d7c%, gd6.. |
| Trimipramine | d7f% |
| Mirtazapine | daB% |
| Mianserin | d7b% |
| Trazodone | d7e% |
|  |  |
| Monoamine oxidase inhibitors (MAOIs) | Read Code |
| Phenelzine | d81% |
| Isocarboxazid | d83% |
| Tranylcypromine | d84% |
|  |  |
| Drugs for psychosis and agitation | Read Code |
| Chlorpromazine | d41%, dh2.., o52% |
| Haloperidol | d47%, d55%, ds1.. |
| Fluphenazine | d46%, d52%, d53% |
| Risperidone | d4p% |
| Quetiapine | d4s% |
| Olanzapine | d4r.., d58% |
|  |  |
| Serotonin and norepinephrine reuptake inhibitor (SNRI) antidepressants | Read Code |
| Venlafaxine | da7% |
| Duloxetine | gde% |
|  |  |
| Opiate analgesics | Read Code |
| Opiate analgesics | dj% |
| Codeine | a81%, cg1%, j28O., j28V., j28z., j2pT. |
| Morphine | a84%, cg3%, ch12., ch13., dq9%, gh6%, o45% |
|  |  |
| Anti-epileptics | Read Code |
| Phenytoin | bc6%, dn8%, dn9%, dni%, do6% |
| Carbamazepine | dn3% |
| Phenobarbitone | a517., dn7% |
|  |  |
| Parkinson’s disease (PD): Dopamine agonists | Read Code |
| Ropinirole, | dqA% |
| Pramipexole | dqE% |
|  |  |
| Parkinson’s disease (PD): MAOI-B inhibitors | Read Code |
| Selegiline | dq6% |
|  |  |
|  |  |
| **Psychotropic drugs, medium risk** |  |
| Selective serotonin reuptake inhibitor (SSRI) antidepressants | Read Code |
| Sertraline | da5% |
| Citalopram | da9%, daC% |
| Paroxetine | da6% |
| Fluoxetine | da4% |
|  |  |
| Anti-epileptics | Read Code |
| Sodium valproate | dnb% |
| Gabapentin | dnj% |
|  |  |
| Muscle relaxants | Read Code |
| Baclofen | j82% |
| Dantrolene | j84%, o91% |
|  |  |
|  |  |
| **Psychotropic drugs, possible risk** |  |
| Anti-epileptics | Read Code |
| Lamotrigine | dnf% |
| Pregabalin | dnp% |
| Topiramate | dnk% |
|  |  |
| Vestibular sedatives | Read Code |
| Prochlorperazine | d4d.., dhe% |
| Betahistine | dh1% |
|  |  |
| Sedating antihistamines for allergy | Read Code |
| Chlorphenamine | c84% |
| Promethazine | c8i%, d19.., dhf.., dhg%, o59.. |
|  |  |
| Anticholinergics acting on the bladder | Read Code |
| Oxybutinin | gda% |
| Tolterodine | gdA% |
| Solifenacin | gdd% |
|  |  |
|  |  |
| **Drugs acting on the heart and cardiovascular system, High Risk** |  |
| Alpha receptor blockers | Read Code |
| Doxazosin | bh6% |
| Indoramin | bh1% |
| Prazosin | bh4% |
| Tamsulosin | gc7%, gc8% |
|  |  |
|  |  |
| Centrally acting alpha 2 receptor agonists | Read Code |
| Clonidine | bf1% |
| Moxonidine | bf4% |
|  |  |
| Thiazide diuretics | Read Code |
| Bendroflumethiazide | b21% |
| Chlorthalidone | b23%, b91e. |
| Metolazone | b2b% |
|  |  |
| Angiotensin converting enzyme inhibitors (ACEIs) | Read Code |
| Lisinopril | bi3% |
| Ramipril | bA1%, bi6% |
| Enalapril | bi2% |
| Captopril | bi1% |
| Perindopril | bi5%, biC% |
| Fosinopril | bi7% |
| Trandolapril | bi9%, bk6% |
| Quinapril | bi4% |
|  |  |
| Beta blockers | Read Code |
| Atenolol | bd3% |
| Sotalol | bdc% |
| Bisoprolol | bdf% |
| Metoprolol | bd6% |
| Propranolol | bd1%, bdn% |
| Carvedilol | bdl% |
| Timolol | bdd%, k8f%, k8k%, k8m%, k8p%, k8q%, k8r%, k8s% |
|  |  |
| Antianginals | Read Code |
| Glyceryl trinitrate (GTN) | ak2%, bl1%, blz% |
| Isosorbide mononitrate | bl3%, blk%, blm% |
| Nicorandil | blf% |
|  |  |
|  |  |
| **Drugs acting on the heart and circulation, medium risk** |  |
| Loop diuretics | Read Code |
| Furosemide | b31%, b51H., b51J., b91f., b91g. |
| Bumetanide | b32.., b51D., b91b. |
|  |  |
| Angiotensin receptor blockers (ARBs) | Read Code |
| Losartan | bk3% |
| Candesartan | bk7% |
| Valsartan | bk4%, bkD% |
| Irbesartan | bk5% |
| Olmesartan | bkB%, bkC%, bkH%, bkI% |
| Eprosartan | bk9% |
|  |  |
| Calcium channel blockers | Read Code |
| Amlodipine | bkD%, blb% |
| Felodipine | blc% |
| Nifedipine | bl8%, bll% |
| Lercanidipine | blh% |
| Diltiazem | bl5%, blj% |
| Verapamil | bb3% |
|  |  |
| Other antidysrhythmics | Read Code |
| Digoxin | b11%, b14% |
| Amiodarone | bb1% |
| Flecainide | bc3% |
|  |  |
|  |  |
| **Drugs acting on the heart and circulation, possible risk** |  |
| Acetylcholinesterase inhibitors | Read Code |
| Donepezil | dy1% |
| Rivastigmine | dy2% |
| Galantamine | dy3% |
|  |  |
