# Supplementary material for: Predicting Falls and When to Intervene in Older People: A Multilevel Logistical Regression Model and Cost Analysis
Source: PLoS One. 2016 Jul 22;11(7):e0159365. doi: 10.1371/journal.pone.0159365 (PMC4957756; doi:10.1371/journal.pone.0159365)
Supplement: S3 Table — (DOCX) [file pone.0159365.s004.docx]

**Appendix 2 – List of variables**

| \| Variable \| Definition \| \| --- \| --- \| \| GenderCode \| Gender \| \| AgeGroup \| Age group \| \| GPPracticeCode \| GP practice \| \| MDG \| Multidisciplinary Group \| \| CCG \| Care Commissioning Group \| \| IMD \| Index of Multiple Deprivation \| \| DiagCnt2_flg \| 2 distinct in-patient primary diagnosis (any episode) \| \| DiagCnt3_flg \| 3 distinct in-patient primary diagnosis (any episode) \| \| DiagCnt4pl_flg \| 4+ distinct in-patient primary diagnosis (any episode) \| \| IP_util_E1pl_m01_flg \| Most recent hospital inpatient episode in the 30 days prior to index date \| \| IP_util_E1_m02_flg \| Most recent hospital inpatient episode 31 to 60 days prior to index date \| \| IP_util_E2pl_m02_flg \| ≥ 2 hospital inpatient episodes 2 months prior to index date \| \| IP_util_E1_m03_flg \| 1 hospital inpatient episode 3 months prior to index date \| \| IP_util_E1_m06_flg \| 1 hospital inpatient episode 6 months prior to index date \| \| IP_util_E2_m06_flg \| 2 hospital inpatient episodes 6 months prior to index date \| \| IP_util_E3pl_m06_flg \| ≥3 hospital inpatient episodes 6 months prior to index date \| \| IP_util_E1_m12_flg \| 1 hospital inpatient episode 12 months prior to index date \| \| IP_util_E2_m12_flg \| 2 hospital inpatient episodes 12 months prior to index date \| \| IP_util_E3pl_m12_flg \| ≥3 hospital inpatient episodes 12 months prior to index date \| \| IP_util_EpisperE3pl_flg \| ≥3 hospital inpatient episodes prior to index date \| \| IP_HospOE \| Observed/Expected ratio for rate of rehospitalisation \| \| OP_NumVisit1_m01_flg \| 1 outpatient visit 1 month prior to index date \| \| OP_NumVisit2_m01_flg \| 2 outpatient visits 1 month prior to index date \| \| OP_NumVisit3pl_m01_flg \| ≥3 Outpatient visits 1 month prior to index date \| \| OP_NumVisit1_m02_flg \| 1 outpatient visit 2 months prior to index date \| \| OP_NumVisit2_m02_flg \| 2 outpatient visits 2 months prior to index date \| \| OP_NumVisit3pl_m02_flg \| ≥3 Outpatient visits 2 months prior to index date \| \| OP_NumVisit0105_m12_flg \| 1-5 outpatient visits 12 months prior to index date \| \| OP_NumVisit0610_m12_flg \| 6-10 outpatient visits 12 months prior to index date \| \| OP_NumVisit11pl_m12_flg \| ≥11 outpatient visits 12 months prior to index date \| \| OP_Thoracic_m12_flg \| Thoracic outpatient visit 12 months prior to index date \| \| OP_Nephro_m12_flg \| Nephrology outpatient visit 12 months prior to index date \| \| OP_TandO_m12_flg \| Orthopaedic outpatient visit 12 months prior to index date \| \| OP_numb3to5_m12_flg \| 3-5 outpatient attendances during last 12 months \| \| AE_Invst01_m03_flg \| A+E investigation 3 months prior to index date \| \| AE_ArrAmb_m02_flg \| A+E arrival by ambulance 2 months prior to index date \| \| AE_NumVisit1_m06_flg \| 1 A+E visit 6 months prior to index date \| \| AE_NumVisit2_m06_flg \| 2 A+E visits 6 months prior to index date \| \| AE_NumVisit3pl_m06_flg \| ≥3 A+E visits 6 months prior to index date \| \| GP_dis47_y12 \| Psychoactive substance misuse disorder \| \| Creatin_3_y02 \| Glomerular Filtration Rate Group 3 \| \| GP_POLY_0104_123 \| 1-4 unique drugs last 0-90 days \| \| GP_POLY_0509_123 \| 5-9 unique drugs last 0-90 days \| \| GP_POLY_10pl_123 \| Polypharmacy ≥10 drugs \| \| ASTHMA_LTC \| Asthma (long term condition) \| \| COPD_LTC \| Asthma (long term condition) \| \| DEPR_LTC \| Depression (long term condition) \| \| CAD_LTC \| Coronary artery disease (long term condition) \| \| DIAB_LTC \| Diabetes (long term condition) \| \| CNCR_LTC \| Cancer (long term condition) \| \| ChrCnt3_flg \| Diagnosed with 3 chronic conditions \| \| ChrCnt4pls_flg \| Diagnosed with 4 or more chronic conditions \| \| MH_LTC \| Mental health (long term condition) \| \| Epilepsy_LTC \| Epilepsy (long term condition) \| \| smoker \| Smoker \| \| stroke \| Previous stroke \| \| multiple_sclerosis \| Multiple sclerosis \| \| statins1 \| Takes a statin \| \| vulnerable_people \| Vulnerable person flag \| \| UTI60 \| Urinary tract infection \| \| NELAdmInPreYear \| Non-elective (NEL) admission in the year prior to index date \| \| NumNELAdmInPreYear \| Number of NEL admissions in the year prior to index date \| \| NELAdmPost30Days \| NEL admission 30 days after the index date \| \| NumNELAdmPost30Days \| Number of NEL admissions in the 30 days after the index date \| \| NELTariffPost30Days \| NEL admissions PbR tariff 30 days following the index date \| \| NELAdmPostYear \| NEL admission in the year following the index date \| \| NumNELAdmPostYear \| Number of NEL admissions in the year following the index date \| \| NELTariffPostYear \| NEL admissions PbR tariff 1 year following the index date \| \| SUSFractureFlagPreany \| Hospital code of fragility fracture prior to index date \| \| SUSFractureFlagPre12 \| Hospital code of fragility fracture 12 months prior to index date \| \| SUSFractureFlagPre6 \| Hospital code of fragility fracture 6 months prior to index date \| \| GPFractureFlagPreany \| GP coding of fragility fracture prior to index date \| \| GPFractureFlagPre12 \| GP coding of fragility fracture 12 months prior to index date \| \| GPFractureFlagPre6 \| GP coding of fragility fracture 6 months prior to index date \| \| SUSFractureFlagPostany \| Hospital code of fracture following the index date \| \| SUSFractureFlagPost6 \| Hospital code of fracture 6 months following the index date \| \| GPFractureFlagPostany \| GP code of fracture following the index date \| \| GPFractureFlagPost6 \| GP code of fracture 6 months following the index date \| \| GPOsteoFlag \| Osteoporosis \| \| Psychotropic_Red_Flag \| Psychotropic red flag drug (see additional list) \| \| Psychotropic_Amber_Flag \| Psychotropic amber flag drug (see additional list) \| \| Psychotropic_Yellow_Flag \| Psychotropic yellow flag drug (see additional list) \| \| HeartMed_Red_Flag \| Cardiovascular red flag drug (see additional list) \| \| HeartMed_Amber_Flag \| Cardiovascular amber flag drug (see additional list) \| \| HeartMed_Yellow_Flag \| Cardiovascular yellow flag drug (see additional list) \| \| GPFallsFlagPreany \| GP coding of previous fall prior to index date date \| \| GPFallsFlagPre12 \| GP coding of fall 12 months prior to index date \| \| GPFallsFlagPre6 \| GP coding of fall 6 months prior to index date \| \| GPFallsFlagPostany \| GP coding of previous fall after index date \| \| GPFallsFlagPost12 \| GP coding of fall 12 months after index date \| \| GPFallsFlagPost6 \| GP coding of fall 6 months after index date \| \| SUSFallsFlagPreany \| Hospital code of a fall prior to the index date \| \| SUSFallsFlagPre12 \| Hospital code of a fall 12 months prior to the index date \| \| SUSFallsFlagPre6 \| Hospital code of a fall 6 months prior to the index date \| \| SUSFallsFlagPostany \| Hospital code of a fall following the index date \| \| SUSFallsFlagPost12 \| Hospital code of a fall 12 months following the index date \| \| SUSFallsFlagPost6 \| Hospital code of a fall 6 months following the index date \| \| NocturiaFlag \| Nocturia \| \| GPOsteoarthritisFlag \| Osteoarthritis \| \| GPOsteoporosisFlag \| Osteoporosis \| \| SUSFractureFlagPost12 \| Hospital code of a fracture 12 months following index date \| \| GPFractureFlagPost12 \| GP code of a fracture 12 months following the index date \| \| postFrac \| GP or Hospital fracture any time following the index date \| \| postFall \| GP or Hospital fall any time following the index date \| \| postFallFrac \| GP or Hospital fall or fracture and time following the index date \| \| NumNELAdmInPreYearCap5 \| Number of non-elective admissions capped at 5 \| \| IMDgrouped \| Index of multiple deprivation grouped into quintiles \| \| FallsFlagPreany \| GP or Hospital fall any time prior to the index date \| \| FallsFlagPre6 \| GP or Hospital fall 6 months prior to the index date \| \| FallsFlagPre12 \| GP or Hospital fall 12 months prior to the index date \| \| postFracHosp \| Hospital fracture any time following the index date \| \| postFallHosp \| Hospital fall any time following the index date \| \| postFallFracHosp \| Hospital fall or fracture any time following the index date \| |  |
| --- | --- | --- | --- | --- | --- | --- | --- | --- | --- | --- | --- | --- | --- | --- | --- | --- | --- | --- | --- | --- | --- | --- | --- | --- | --- | --- | --- | --- | --- | --- | --- | --- | --- | --- | --- | --- | --- | --- | --- | --- | --- | --- | --- | --- | --- | --- | --- | --- | --- | --- | --- | --- | --- | --- | --- | --- | --- | --- | --- | --- | --- | --- | --- | --- | --- | --- | --- | --- | --- | --- | --- | --- | --- | --- | --- | --- | --- | --- | --- | --- | --- | --- | --- | --- | --- | --- | --- | --- | --- | --- | --- | --- | --- | --- | --- | --- | --- | --- | --- | --- | --- | --- | --- | --- | --- | --- | --- | --- | --- | --- | --- | --- | --- | --- | --- | --- | --- | --- | --- | --- | --- | --- | --- | --- | --- | --- | --- | --- | --- | --- | --- | --- | --- | --- | --- | --- | --- | --- | --- | --- | --- | --- | --- | --- | --- | --- | --- | --- | --- | --- | --- | --- | --- | --- | --- | --- | --- | --- | --- | --- | --- | --- | --- | --- | --- | --- | --- | --- | --- | --- | --- | --- | --- | --- | --- | --- | --- | --- | --- | --- | --- | --- | --- | --- | --- | --- | --- | --- | --- | --- | --- | --- | --- | --- | --- | --- | --- | --- | --- | --- | --- | --- | --- | --- | --- | --- | --- | --- | --- | --- | --- | --- | --- | --- | --- | --- | --- | --- | --- | --- | --- | --- | --- | --- | --- | --- | --- | --- | --- |
|  |  |
|  |  |
|  |  |
|  |  |
|  |  |
|  |  |
|  |  |
|  |  |
|  |  |
|  |  |
|  |  |
|  |  |
|  |  |
