# Supplementary material for: Predicting Falls and When to Intervene in Older People: A Multilevel Logistical Regression Model and Cost Analysis
Source: PLoS One. 2016 Jul 22;11(7):e0159365. doi: 10.1371/journal.pone.0159365 (PMC4957756; doi:10.1371/journal.pone.0159365)
Supplement: S4 Table — (DOCX) [file pone.0159365.s005.docx]

**Appendix 3- Alternative cost analysis**

Several values for the cost of a fall were modelled for the cost analysis calculations.

1. Initially a value from a Kingsfund paper, estimating the immediate cost of a fall and the increased healthcare costs over the following 12 months at a combined £11442 [38]. This value was initially applied to all falls from GP and Hospital data.
2. The same value was then applied to falls coded in hospital data only, as the Kingsfund figure related to hospitalised falls. This was more valid however it was suspected that the value was still an overestimate.
3. Next, lower estimates of the cost of a fall dependent on the age of the patient, from a study carried out in York were used, again applied to hospital falls only [39]. This was thought to be more representative of the actual cost of a fall.
4. Finally the exact figures from the additional health utilisation in our falling population compared to non-fallers was used. This was thought to be the most valid as it represents the actual additional costs, as opposed to estimates of the cost.

**Table: Different cost models used, and their effects on the cut-off value at which savings are made, and the implications of this on referral numbers.**

| Model | Saving  cut-off | (95% CI) | % of population referred | (95% CI) | % of total falls in referred population | (95% CI) |
| --- | --- | --- | --- | --- | --- | --- |
| Hospital and GP falls, Cost of fall £11442 | 0.07 | (0.03, 0.28) | 22% | (44%, 5%) | 77% | (91%, 39%) |
| Hospital falls only, Cost of fall £11442 | 0.09 | (0.04, 0.42) | 7% | (17%, 0.8%) | 48% | (69%, 12%) |
| Hospital falls only, York costs of fall | 0.64 | (0.32, 0.86) | 0.19% | (1.4%, 0.01%) | 0.04% | (18%, 0.003%) |
| Hospital falls only, PbR cost of fall | 0.27 | (0.11, 0.51) | 1.8% | (5.8%, 0.5%) | 22% | (43%, 8%) |
