# Supplementary material for: Predicting Falls and When to Intervene in Older People: A Multilevel Logistical Regression Model and Cost Analysis
Source: PLoS One. 2016 Jul 22;11(7):e0159365. doi: 10.1371/journal.pone.0159365 (PMC4957756; doi:10.1371/journal.pone.0159365)
Supplement: S5 Table — (DOCX) [file pone.0159365.s006.docx]

**Appendix 4 – Comparison of risk model with existing models**

Table comparing our falls risk model to existing models and tools:

**Table: Comparison of our falls risk model (top) with existing falls models.** The models are ordered by AUC of the ROC curve where it was described in the literature, and then by year of study.

| Tool/model | Year | Cut off | Sensitivity | Specificity | PPV | NPV | AUC | Youden’s |
| --- | --- | --- | --- | --- | --- | --- | --- | --- |
| Our New falls model | 2014 | ≥0.07 | 0.78 | 0.81 | 0.21 | 0.98 | 0.87 | 0.56 |
| Stalenhoef et al [33] | 2002 | ≥0.30 | 0.59 | 0.87 | 0.52 | 0.90 | 0.79 | - |
| Precursors of falling (PF-3) score [40] | 2012 | ≥1 | 0.87 | 0.65 | - | - | 0.79 | - |
| TMIG-15 score [40] | 2012 | ≥4 | 0.59 | 0.83 | - | - | 0.78 | - |
| Woo et al [41] | 2009 | >8 | 0.56(M)  0.64(F) | 0.78  0.71 | 0.16 | 0.95 | 0.75  0.73 | - |
| Stalenhoef et al [42] | 2000 | ≥0.26 | 0.64 | 0.71 | 0.42 | 0.86 | 0.73 | - |
| FROP-com. [43] | 2009 | ≥4 | 0.67 | 0.66 | 0.64 | 0.68 | 0.73 | - |
| Gunn et al [44] | 2013 | ≥0.51 | 0.70 | 0.69 | - | - | 0.73 | - |
| Our Previous falls model [8] | 2014 | ≥9 | 0.68 | 0.60 | 0.13 | 0.96 | 0.72 | 0.28 |
| Tromp et al [45] | 2001 | ≥7 | 0.54 | 0.79 | 0.25 | 0.93 | 0.71 | - |
| Pluijm et al [46] | 2006 | 5/30  10/30 | 0.59  0.31 | 0.71  0.92 | 0.39 | 0.85 | 0.71 | - |
| PROFET [47] | 1999 | - | - | - | - | - | 0.70 | - |
| Bongue et al [48] | 2011 | 3/12  7/12 | 0.70  0.19 | 0.60  0.96 | 0.45  0.72 | 0.81  0.72 | 0.70 | - |
| DFRA score [40] | 2012 | ≥22 | 0.31 | 0.07 | - | - | 0.68 | - |
| Tiedemann et al [49] | 2013 | 2+ falls  6+ med | - | - | - | - | 0.67 | - |
| BERG Balance test [50] | 2013 | <32 | 0.51 | 0.57 | - | - | 0.64 | - |
| One-leg standing [51] | 2004 | - | - | - | - | - | 0.61 | - |
| Timed Up And Go [52] | 2014 | ≥13.5s | 0.32 | 0.73 | - | - | 0.57 | - |
| Tinetti Mobility score (POMA) [51] | 2004 | - | - | -- | - | - | 0.56 | - |
| Functional reach [51] | 2004 | - | - | - | - | - | 0.51 | - |
| STRATIFY [53] | 1997 | ≥2 | 0.93 | 0.88 | 62.3 | 98.3 | - | - |
| Hendrich II score [54] | 2003 | ≥5 | 0.75 | 0.74 | - | - | - | - |
| Innes score [7] | 2004 | - | 89.3 | 73.5 | 7.3 | 99.7 | - | - |
| Morse score [7] | 2004 | ≥45 | 73.2 | 75.1 | 4.3 | 99.4 | - | - |
| Downton score [7] | 2004 | ≥3 | 90.6 | 26.8 | 44.4 | 81.5 | - | - |
| Schmid score [7] | 2004 | ≥3 | 92.5 | 78.2 | 36.6 | 98.7 | - | - |
| Falls Risk Assessment Tool (FRAT) [5] | 2004 | ≥2  ≥3  ≥3 | 0.59  0.42  0.15 | 0.80  0.92  0.97 | 0.43  0.57  0.58 | 0.88  0.86  0.82 | - | 0.39  0.34  0.13 |
